# Visualization and Quantification of Post-stroke Neural Connectivity and Neuroinflammation Using Serial Two-Photon Tomography in the Whole Mouse Brain

**DOI:** 10.3389/fnins.2019.01055

**Published:** 2019-10-04

**Authors:** Katherine Poinsatte, Dene Betz, Vanessa O. Torres, Apoorva D. Ajay, Shazia Mirza, Uma M. Selvaraj, Erik J. Plautz, Xiangmei Kong, Sankalp Gokhale, Julian P. Meeks, Denise M. O. Ramirez, Mark P. Goldberg, Ann M. Stowe

**Affiliations:** ^1^Department of Neurology and Neurotherapeutics, UT Southwestern Medical Center, Peter O'Donnell Jr. Brain Institute, Dallas, TX, United States; ^2^Department of Neuroscience, UT Southwestern Medical Center, Dallas, TX, United States; ^3^Department of Neurology, University of Kentucky, Lexington, KY, United States

**Keywords:** stroke, serial two-photon tomography, whole brain imaging, neural connectivity, neuroinflammation, CD8 T cells, pseudorabies virus, corticospinal tract

## Abstract

Whole-brain volumetric microscopy techniques such as serial two-photon tomography (STPT) can provide detailed information on the roles of neuroinflammation and neuroplasticity throughout the whole brain post-stroke. STPT automatically generates high-resolution images of coronal sections of the entire mouse brain that can be readily visualized in three dimensions. We developed a pipeline for whole brain image analysis that includes supervised machine learning (pixel-wise random forest models via the “ilastik” software package) followed by registration to a standardized 3-D atlas of the adult mouse brain (Common Coordinate Framework v3.0; Allen Institute for Brain Science). These procedures allow the detection of cellular fluorescent signals throughout the brain in an unbiased manner. To illustrate our imaging techniques and automated image quantification, we examined long-term post-stroke motor circuit connectivity in mice that received a motor cortex photothrombotic stroke. Two weeks post-stroke, mice received intramuscular injections of pseudorabies virus (PRV-152), a trans-synaptic retrograde herpes virus driving expression of green fluorescent protein (GFP), into the affected contralesional forelimb to label neurons in descending tracts to the forelimb musculature. Mice were sacrificed 3 weeks post-stroke. We also quantified sub-acute neuroinflammation in the post-stroke brain in a separate cohort of mice following a 60 min transient middle cerebral artery occlusion (tMCAo). Naive e450^+^-labeled splenic CD8^+^ cytotoxic T cells were intravenously injected at 7, 24, 48, and 72 h post-tMCAo. Mice were sacrificed 4 days after stroke. Detailed quantification of post-stroke neural connectivity and neuroinflammation indicates a role for remote brain regions in stroke pathology and recovery. The workflow described herein, incorporating STPT and automated quantification of fluorescently labeled features of interest, provides a framework by which one can objectively evaluate labeled neuronal or lymphocyte populations in healthy and injured brains. The results provide region-specific quantification of neural connectivity and neuroinflammation, which could be a critical tool for investigating mechanisms of not only stroke recovery, but also a wide variety of brain injuries or diseases.

## Introduction

In the United States, nearly 800,000 people have a stroke annually, making it the 5th leading cause of death and the leading cause of adult disability (Benjamin et al., 2018). More than half of all stroke survivors experience persistent motor deficits that impair daily function, underscoring the significant need for a better understanding of the mechanisms of injury and repair after stroke (Dobkin, 2005). Spontaneous recovery of function is associated with a higher capacity for neuroplasticity in homologous brain regions after stroke (Brown et al., 2009). These regions, which are both remote and adjacent to the infarcted tissue, undergo changes in order to assume the functions of the lost neuronal networks (Furlan et al., 1996). Preclinical models of stroke have been instrumental in understanding the complexity of post-stroke responses and how they relate to functional recovery (Nudo, 2007). Using these models, plasticity which underlies circuit remodeling has been repeatedly shown to be correlated with improved recovery from stroke (Jones and Adkins, 2015). These forms of circuit plasticity involve recruitment of homologous neuronal pathways, inhibition of aberrant electrical signaling, and formation of new connections through synaptogenesis and axonal sprouting throughout the brain and spinal cord (Murphy and Corbett, 2009). However, in some cases, post-stroke neuroplasticity can impede recovery. The degree of functional recovery is heavily dependent on the regions in which the plasticity occurs (Dancause et al., 2005; Kim et al., 2005), emphasizing the need for pre-clinical studies that investigate changes in axonal connectivity in the whole brain after stroke.

In addition to alterations in brain architecture after stroke, the immune system plays a major role in post-stroke pathology and recovery. In particular, the critical role of the immune system in both the acute and chronic phases of post-stroke recovery is becoming increasingly apparent (Jin et al., 2013). Within hours of the onset of injury, damage-associated molecular patterns (DAMPS) are released from dying neurons, astrocytes, and endothelial cells, recruiting immune cells to the brain (Rubartelli and Lotze, 2007). Blood-brain barrier (BBB) disruption, upregulation of cell adhesion molecules, and activation of resident microglia enhance the post-stroke neuro-immune interactions (Abulrob et al., 2008; Patel et al., 2013). Within 24 h of stroke onset, adaptive immune cells, including T cells, can be detected in the ipsilesional hemisphere (Gelderblom et al., 2009). Migration of these cells peaks around 3–4 days post-stroke, but helper CD4^+^ and cytotoxic CD8^+^ T cells persist in the perilesional tissue for weeks after injury, implicating this leukocyte subset in long-term recovery (Xie et al., 2018). Many of these studies rely on flow cytometry, a technique that requires the dissociation of brain tissue, and therefore the loss of information of spatial dynamics of leukocyte diapedesis. Even research using spatially-sensitive techniques like immunohistochemistry often overlook the possibility of recruitment of immune cells into remote brain areas. Given that leukocytes modulate axonal growth after injury through the secretion of cytokines and growth factors (Wang et al., 2018), it is possible that migration of immune cells, such as CD8^+^ T cells, to remote brain regions could affect post-stroke plasticity far from the stroke lesion. This highlights the need to understand where neuroplasticity and neuroinflammation occur in the brain after stroke, and if the two phenomena are interconnected. To visualize post-stroke responses that directly involve neuroplasticity or occur on the larger scale, systematic analysis of the entire brain must be incorporated into preclinical studies of stroke recovery.

Advances in the field of functional imaging have greatly enhanced the ability to study global post-stroke alterations in brain connectivity (Bauer et al., 2014). However, few studies currently exist which incorporate the use of high resolution, whole brain volumetric microscopy techniques to assess changes in brain architecture in the context of stroke (Lugo-Hernandez et al., 2017). In the current study, we used serial two-photon tomography (STPT) (Ragan et al., 2012; Oh et al., 2014; Amato et al., 2016; Kim et al., 2016; Whitesell et al., 2018; Ramirez et al., 2019), a block-face automated imaging technique, to acquire volumetric image datasets comprising entire mouse brains. A custom-developed image analysis pipeline incorporating supervised machine learning (ML)-based methods to isolate relevant fluorescent signals of interest and registration of the images into the Allen Institute Common Coordinate Framework version 3.0 (CCF 3.0) was then employed to quantify stroke-induced neural connectivity and neuroinflammation throughout the mouse brain. We found robust changes in connectivity in the corticospinal tract, as well as CD8^+^ T cell migration into motor and somatosensory brain regions affected by stroke. Taken together, our results indicate that mesoscale whole-brain imaging reveals important information about region-specific changes in both neural connectivity and neuroinflammation that may be key to developing long-term therapies to promote functional recovery after stroke.

## Methods

### Animal Approval

Experiments involving corticospinal tract tracing with PRV-152 used 8–11-week old adult, male C57BL/6 (wild type; WT) mice from Jackson Laboratories. For neuroinflammation experiments involving CD8^+^ T cells, 10–15-week-old male WT mice were randomized for the isolation of donor CD8^+^ T cells or to receive the donor T cells following stroke. Animals were housed in a 12 h light/dark cycle with access to food and water *ad libitum*. All protocols were approved by the University of Texas Southwestern Medical Center Institutional Animal Care and Use Committee.

### Photothrombotic (PT) Stroke

Cortical stroke was induced using the photothrombotic stroke model previously described (Yanev et al., 2019). Mice (*n* = 6) were anesthetized using 1–4% isoflurane, 0.7% nitric oxide, and 0.3% oxygen and temperature and breathing rates were monitored. Mice were placed on a stereotaxic frame and an incision was made down the midline of the scalp. Mice were administered 1.5 mg of Rose Bengal (Sigma Aldrich, St. Louis, MO, USA) dissolved in 0.3 cc of saline via intraperitoneal injection. One minute later, we aimed a 45 mW laser (Coherent Sapphire, Santa Clara, CA, USA; 561 nm; 2.7 mm collimated beam diameter) 1.7 mm lateral to Bregma as the forelimb representation of the motor cortex for 15 min. Buprenorphine was administered post-operation for pain management, and moist food was provided for the first 24 h following stroke. All mice that received a PT stroke had a successful surgery.

### Transient Middle Cerebral Artery Occlusion (tMCAo)

Mice (*n* = 7) were anesthetized (2% isoflurane/ 70% NO_2_/30% O_2_) and their body temperatures were maintained at 37°C while the left middle cerebral artery (MCA) was exposed for transcranial Laser Doppler flowmetry (TSI, Inc.) as previously described (Monson et al., 2014; Ortega et al., 2015). A blunted suture (6.0-gauge nylon, 12 mm) was advanced to block the MCA (>80% reduction relative to baseline blood flow) by surgeons blinded to condition, between 8 and 14:00 h. Animals were placed in an incubator (34°C), re-anesthetized after 60 min, and suture withdrawn. Flowmetry confirmed reperfusion (CBF > 50% baseline) and animals were monitored. All animals met blood flow criteria and were included in analysis.

### Intramuscular Injections of Pseudorabies Virus (PRV)

An incision was made on ulnar border of forelimb on anesthetized mice using a standard scalpel (Liu et al., 2009). After the forelimb flexor muscle was identified, mice were given an intramuscular injection using a 27 G needle and 10 μL syringe of PRV-152, a generous gift from Dr. Lynn Enquist (Princeton University, Princeton, NJ), a trans-synaptic pseudorabies virus expressing green fluorescent protein (GFP). PRV-152 injections were divided into multiple injections of 2 μL of virus in multiple locations in the forelimb flexor muscle (Ganzer et al., 2018). All standard viral handling precautions were followed during this procedure. Animals recovered in a standard recovery chamber. A single administration (0.2 ml) of subcutaneous buprenorphine (0.05 mg/kg) and lidocaine was given immediately after the procedure for pain relief. Mice that were injected with PRV that received a stroke had a 50% morality rate (3/6 mice) while mice that received a sham surgery all survived (6/6 mice) to the 6-day sacrifice time point.

### Sample Preparation for Imaging on TissueCyte 1000

All mice were transcardially perfused with 1X Phosphate Buffered Saline (PBS) followed by 4% paraformaldehyde (PFA) to fix brain tissues. Extracted tissues were further post-fixed in 4% PFA at 4°C for 24 h and transferred to 0.01% NaN_3_ in 1X PBS for storage at 4°C until embedding. 4.5% (w/v) agarose (Type 1A, low EEO, Sigma #A0169) solution in 50 mM phosphate buffer was first prepared and oxidized by adding 10 mM NaIO_4_ (Sigma #S1878) while stirring gently for 2 to 3 h in the dark. The agarose solution was filtered with vacuum suction and washed 3 times with 50 mM phosphate buffer. The washed agarose was resuspended in the appropriate volume of phosphate buffer to make a 4.5% agarose solution. The oxidized agarose solution was heated to boiling in a microwave, then transferred to a stirring plate and allowed to cool to 60–65°C. Brains were embedded by filling a cryoembedding mold (VWR #15560-215) with oxidized agarose, placing the filled mold on a flat frozen ice pack, and then quickly submerging the brain using forceps into the bottom of the block with the cerebellum touching the bottom of the block (olfactory bulbs facing upward). The agarose block was allowed to fully solidify on a frozen ice pack in the dark. Once the agarose blocks were fully hardened, the agarose blocks including the specimens were removed from the molds and placed in small glass jars, where they were treated overnight at 4°C in the dark in sodium borohydride buffer (50 mM sodium borohydride, 50 mM borax, 50 mM boric acid, pH 9.0–9.5). After overnight crosslinking, the agarose blocks were transferred to phosphate buffer for storage at 4°C until TissueCyte imaging.

### Serial Two-Photon Tomography

The agarose blocks containing the brain samples were attached to a custom magnetic slide with superglue and placed on a magnetized stage within an imaging chamber filled with phosphate buffer. STPT imaging is a block-face imaging technique in which a series of 2-dimensional (2-D) mosaic images in the coronal plane is acquired just below the cut surface of the brain (within ~100 μm), followed by physical sectioning with a built-in vibrating microtome to cut away the imaged tissue, preparing a new cut surface for imaging (Ragan et al., 2012). For this study, three optical planes were imaged at 25, 50, and 75 μm below the cut surface, followed by a vibrating microtome cut at 75 μm (blade vibration frequency of 70 Hz, advancement velocity 0.5 mm/s). The excitation laser (MaiTai DeepSee, SpectraPhysics/Newport, Santa Clara, CA) wavelength was tuned to either 920 nm to excite GFP (PRV experiments) or 850 nm to excite e450 (CD8^+^ T cell experiments). Three image channels of emission fluorescence (pre-set bandpass filters encompassing red, green, and blue fluorophores) were collected with a predetermined photomultiplier tube voltage of 700 V. This process produced 190 physical sections and 570 2-D stitched coronal section images (9 by 13 mosaic collected for each coronal section) with a lateral resolution of 0.875 μm/pixel and axial resolution of 25 μm (~280 gigabytes of raw data per brain). The raw image tiles were first trimmed and subjected to flat field correction, then stitched into 2-D mosaic coronal section images using the Autostitcher software (TissueVision, Inc.).

### Image Analysis Pipeline

**Figure 1:**
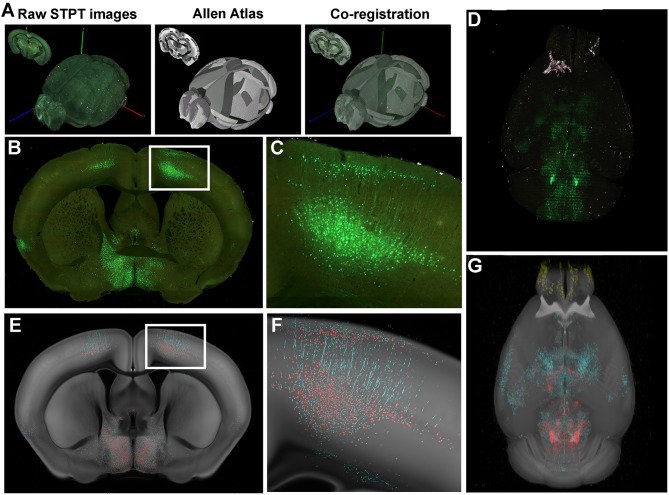
Serial two-photon tomography of the whole mouse brain. **(A)** Overview of serial two-photo tomography (STPT) data acquisition and registration. **(B)** Representative coronal section raw fluorescence image, with **(C)** expansion of areas identified in white box in B showing GFP+ neuronal cell bodies and processes. **(D)** Coronal images are stacked into a 3D representation and co-registered to the Common Coordinate Framework (CCF 3.0; Allen Institute for Brain Science) Atlas. **(E)** Probability map output of the same section shown in **(B)** depicting pixels classified via machine learning (ML) as either GFP+ cell bodies (red) or GFP+ neuronal processes (cyan) overlaid onto the corresponding section from the atlas (CCF 3.0 average template) shown in gray. **(F)** Enlarged image of boxed area in **(E)** showing fine detail and accurate classification of neuronal cell bodies and processes corresponding to the raw fluorescence image shown in **(C)**. **(G)** 3-D rendering of the probability maps corresponding to the raw fluorescence images of the brain shown in D. GFP^+^ cell bodies, red, GFP^+^ neuronal processes, cyan, atlas images, gray.

We used an automated pipeline (as described in Ramirez et al., 2019) to analyze and extract quantitative results from raw STPT-generated data. The image analysis pipeline is depicted in Figure 1 and consists of three main components: image registration, pixel classification and numerical quantification. The red channel 2-D stitched images are used to register the dataset into the Allen Institute Common Coordinate Framework (CCF 3.0) using SimpleElastix (an extension of SimpleITK that combines elastix and transformix) (Lowekamp et al., 2015). A multi-step registration process was chosen using rigid, affine, and b-spline registration components. At each step, optimization was performed on a 6-resolution multi-grid (pyramid) schedule, with Mattes Mutual Information as the optimization metric. To balance local/global optimization across the whole dataset, the “Random Coordinate” Image Sampler was used with sample regions of 2 × 2 × 4 mm. Final b-spline grid dimensions were 0.5 × 0.5 × 0.5 mm. The optimized registration parameters identified for the red channel images were then applied to the other raw image channels and ilastik probability maps using transformix.

Relevant signals of interest were identified in the images using the “Pixel Classification” plugin for the Interactive Learning and Segmentation Toolkit (ilastik), which implements a random forest supervised machine learning (ML) classifier (Somner et al., 2011). To support the supervised ilastik training, a maximum intensity projection (MIP) was first created for each physical section from its three optical planes and the resultant MIP image was downsampled to a pixel size of 1.5 μm × 1.5 μm in the x-y dimension. This resulted in a set of 16-bit 3-color compressed TIFF images with the same number of images as physical sections in the STPT image (190). At least three representative sections containing examples of fluorescent signals of interest (e.g., GFP^−^ expressing neurons or e450-labeled T cells) were selected from each sample. The random forest ML model was trained on these selected sections by assigning labels including as fluorescent cell bodies, tissue autofluorescence, stroke, and other features (e.g., microbubbles surrounding the olfactory bulbs). After completing the initial training, each of the sample images was iteratively updated until visible misclassifications were minimized. Each cohort of brains (PRV injected or T cell injected) was used to train an independent model for classification, and every section from every brain in the cohort was subjected to the same model. Ilastik predictions were exported as an 8-bit “probability map” TIFF for each trained label, with the pixel value of 0 mapping to 0% probability and 255 100% probability of a pixel belonging to the label. The resulting TIFF stacks were warped to the CCF 3.0 using the transformation parameters identified by elastix via transformix. The summed intensity of all voxels lying within CCF 3.0 annotated regions were quantified using custom MATLAB software, producing a matrix of signal intensity for each sample, label, and brain region.

### Image Analysis Validation

To assess the performance of the ilastik-based image analysis pipeline, three independent observers (i.e., different than the individual that trained the ilastik model) selected at least 8 random 1-megapixel subregions from at least two brains from each experimental condition (8 brains total, 4 from CD8^+^ T cell cohort, 4 from PRV −152 cohort) for manual annotation of critical labels (e.g., fluorescent cell bodies, stroke). Each observer utilized Adobe Photoshop tools to create binary masks for each test label, as well as autofluorescence and black background labels. These observer-generated binary masks were considered the reference set, with the ilastik probability maps as the test set. Custom MATLAB software was used to compare each reference image to the corresponding test image while varying the segmentation thresholds in the test image (i.e., to convert the 8-bit range of the probability map to a complementary binary map). From these comparisons the true positive, false negative, false positive, and false negative rates were calculated for each threshold. These values were calculated on a voxel basis to match the values quantified in these studies. For the critical test labels (fluorescent cell bodies and stroke) the ilastik model achieved very low false positive rates even when a segmentation threshold at 5% of the 8-bit range (CD8^+^ T cell bodies: 0.008 ± 0.001%; PRV GFP^+^ cell bodies: 2.0 ± 0.5%; stroke: 0.10 ± 0.03%; *n* = 8 subregions each from 2, 4, and 4 brains, respectively). In control samples for the CD8^+^ T cell experiments (samples lacking labeled T cells), observers noted rare blue spots in the tissue that roughly resembled the small T cell bodies and annotated them. The false positive rate for T cell-negative samples was even lower than for the labeled T cell samples (0.0006 ± 0.0003%, 8 subregions from 2 brains). True positive rates at these thresholds were not saturated (CD8^+^ T cell bodies: 57.0 ± 4.8%; PRV GFP^+^ cell bodies: 84.6 ± 4.4%; stroke: 48.2 ± 0.5%; 2, 4, and 2 brains, respectively). F1 score (scale 0 to 1 with 1 being best performance), a common measurement of model accuracy, indicated strong performance for PRV GFP^+^ cell bodies and adequate performance for CD8^+^ T cell bodies and stroke voxels (CD8^+^ T cell bodies: 0.72 ± 0.13; PRV GFP^+^ cell bodies 0.90 ± 0.03; stroke 0.73 ± 0.04; *n* = 2, 4, and 2 brains, respectively). The Matthews Correlation Coefficient, an alternative metric (ranging from −1, worse than chance, to 0 for chance, to 1 for optimal), showed similar results to the F1 score (CD8^+^ T cell bodies: 0.63 ± 0.04; PRV GFP^+^ cell bodies: 0.84 ± 0.4; stroke 0.64 ± 0.4, *n* = 2, 4, and 2 brains, respectively).

These validation studies indicate that the ilastik model was conservative (prioritized low false positive rates at the expense of true positives) for the labels quantified in these studies.

In order to further validate the ilastik STPT quantification, we manually counted cell bodies in the primary motor cortex (MOp), primary somatosensory cortex (SSp), and supplemental motor cortex (MOs) in both the right and left hemispheres in STPT sections. We counted cells within a range of 71 coronal slices. The initial slice was identified by the morphology of the hippocampus, size of lateral ventricles, and the presence of the fasiculus retroflexus. The final slice in the range was determined from the size of the olfactory bulb. The images used for manual quantification were identical to those used for automated quantification. We classified a cell as a circular object with GFP signal that was clearly distinguishable from the background. Manual quantification of neuronal processes was not performed.

### CD8^+^ T Cell Isolation, Labeling, and Intravenous Injections

Using magnetic CD8 T cell isolation kits (STEMCELL Cat. 19853), donor CD8 T cells were isolated from the spleens of naïve, adult donor WT mice (*n* = 8). A small aliquot of cells was assessed before and after enrichment by flow cytometry to ensure purity of the isolated CD8 T cells. CD8 T cells were then labeled with a fluorescent proliferation dye according to manufacturer's protocol (e450; eBioscience 65-0842-85) and resuspended in 1XPBS immediately prior to injection. On average, ~3.2 × 10^6^ CD8^+^ T cells were intravenous injected into the tail veins of WT recipient mice. Brains were extracted and further post-fixed overnight in 4% PFA at 4°C, transferred to 1X PBS + 0.01% NaN_3_ and stored at 4°C until embedding. Peripheral migration of T cells into secondary lymphoid organs (i.e., spleen and cervical lymph nodes) was confirmed using flow cytometry. Briefly, single cell suspensions were generated from secondary lymphoid organs, and 1 × 10^6^ cells were used for the fluorescent antibody staining to specific leukocyte (CD45-APC-Cy7) and T cell (TCRβ-711, CD4-PE/Dazzle 594, CD8-APC) markers for 30 min. at 4°C and subsequently washed and fixed in 1% paraformaldehyde. Flow cytometric data were acquired using a BD LSRFortessa using FACS Diva 6.0 software and FlowJo 9.0 software was used to subset leukocytes according to gating strategy shown in Supplementary Figure 1.

### Statistics

Statistical differences were analyzed using unpaired parametric two-sample Student's *t*-test, ratio paired student's *t*-test, two-way repeated measures ANOVA where appropriate and indicated in the text (Graph Pad Prism 6.0). Manual cell counts and ML probability values were correlated using a linear regression analysis (Graph Pad Prism 6.0). Values of *p* < 0.05 were considered significant. All experimenters were blinded to condition, and all animal were randomly assigned to group.

## Results

### Whole Brain Visualization of Post-stroke Neural Connectivity

Previous studies utilizing traditional and viral retrograde tracers demonstrated robust descending connections from the brain to the skeletal muscle (Kerman et al., 2003, 2006; Lee et al., 2007). Furthermore, many studies demonstrate that stroke induces widespread changes in neuronal connectivity. Much attention has been given to remodeling of the corticospinal and corticorubral tracts (Ishida et al., 2016; Okabe et al., 2017), including increased corticospinal innervation of the stroke-impaired forelimb from the uninjured contralesional motor cortex (Bachmann et al., 2014). One recent study showed that connectivity in the brainstem also undergoes dynamic changes after a photothrombotic (PT) stroke in the primary motor cortex, highlighting the importance of global interrogation of circuitry (Bachmann et al., 2014). Imaging techniques, such as *in vivo* magnetic resonance imaging (MRI), can be used in mice to quantify whole-brain changes in connectivity after stroke (Dijkhuizen et al., 2012; Bauer et al., 2014). However, such techniques do not offer sufficient resolution to visualize cell bodies or axonal processes. Microscopy methods such as STPT can visualize and quantify these fine details, making these techniques vital in developing an understanding of connectivity on a mesoscale.

To demonstrate how STPT can be used to elucidate post-stroke changes in neural connectivity, 8–11-week-old C57/B6 mice received either a PT focal stroke in the right primary motor cortex (MOp) or a sham surgery. Two weeks later, mice received an intramuscular left forelimb injection of pseudorabies virus (PRV-152) carrying GFP, an effective trans-synaptic retrograde tracer that labels motor tracks which project to distal musculature (Kerman et al., 2003) Following STPT processing of the brains, the resultant images were analyzed using the custom-developed automated pipeline (Figure 1A). During the process of training the ML-based pixel classification algorithm, the random forest classifier was trained to recognize multiple structures of interest including cell bodies, neuronal processes, and areas of infarction. Figure 1 shows raw STPT data of GFP^+^ cell bodies and processes (Figures 1B–D), as well as the probability maps generated from the STPT data for automated identification of cell bodies and processes in the healthy mouse brain (Figures 1E–G). We also generated 3-D videos of these data from an uninjured brain, with fly-through and rotational videos of raw STPT data (Supplementary Videos 1, 2), as well as fly-through and rotation videos of corresponding probability maps (Supplementary Videos 3, 4).

**Figure 2:**
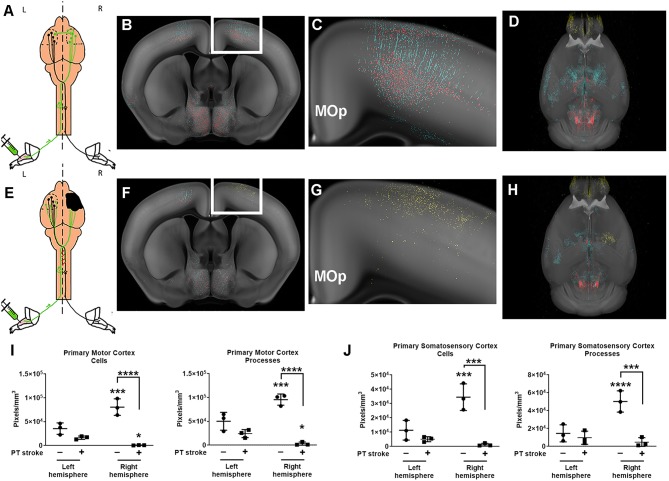
Quantification of changes in forelimb innervation after stroke. **(A)** Schematic of pseudorabies virus (PRV) injection in healthy mice, with **(B)** corresponding coronal section of the probability maps from a representative animal overlaid onto the CCF 3.0 average template (gray) at the level of the primary motor cortex (MOp) **(C)** Enlargement of white boxed area shown in **(B)** depicts pixels classified as cell bodies (red) and neuronal processes (cyan). **(D)** 3-D rendering of the whole brain probability map (images from this animal also shown in Figure 1) **(E)** Schematic of PRV injections after stroke (black area, right hemisphere). **(F)** Corresponding coronal section of the probability maps from a representative post-stroke animal overlaid onto the CCF 3.0 average template (gray) at the level of the primary motor cortex. **(G)** Enlargement of white boxed area shown in **(F)** depicts pixels predominantly classified as infarction (yellow) in the right hemisphere. **(H)** 3-D probability map shows area of infarction in the right hemisphere. **(I,J)** Quantification of cell bodies and processes in **(I)** primary motor cortex (MOp) and **(J)** primary somatosensory cortex (SSp) for both left and right hemispheres of uninjured (circles) and post-stroke (square) cohorts. Data shown are pixels/mm^3^ for each area. *p < 0.05, ***p < 0.001, ****p < 0.0001 vs. left hemisphere unless otherwise indicated by brackets.

The probability maps of these cortical regions show that neuronal cell bodies and their processes in both the left and right motor and somatosensory cortices (SS) of uninjured animals have been accurately identified by the trained ML algorithm, with more cells in the right cortex compared to the left (Figures 2A–D). In addition to the cell bodies and processes of layer 5 neurons, second-order neurons in layer 2 and 3 of the motor cortex and axons passing in the corpus callosum were also visible in labeled data set. The probability maps clearly demonstrate the widespread robust viral labeling, indicating a large number of descending connections to the left forelimb from the predicted motor cortex regions, as well as remote regions in the midbrain and brainstem. In mice that received a stroke, the ML algorithm detected some cells in the contralesional left motor cortex, albeit fewer than observed in uninjured animals (Figures 2E–H). ML classified bright autofluorescent signal in the ipsilesional right motor cortex as infarcted tissue (Figure 2G). While the ML algorithm did not classify all pixels within the lesion as infarcted tissue, it still offered excellent spatial resolution of most of the lesioned tissue and provided detailed visualization of where the PT stroke was induced in the brain (Figure 2H). The 3-D videos of stroke-injured brains clearly identify the PT stroke in both the fly-through and rotational videos of raw STPT data (Supplementary Videos 5, 6), as well as fly-through and rotation videos of corresponding probability maps (Supplementary Videos 7, 8).

In uninjured mice, nearly all cortical sensory and motor areas were labeled in the right hemisphere. Cortical sensory and motor areas had many GFP^+^ cell bodies and processes and therefore, a robust fluorescent signal in these areas in the absence of injury. The highest fluorescent signal was observed in the layer 5 neurons in the right primary motor cortex (MOp5) and in neurons in the primary somatosensory area associated with the lower limb (SSp-II), an area adjacent to the primary motor cortex (Figure 2C). Fewer PRV^+^ cells were detected in left MOp5 neurons and SSp-ll neurons in these uninjured animals, suggesting that there was greater innervation of the left forelimb flexor from the right cortical motor regions in the absence of stroke (Figures 2I,J). After stroke, we observed that there were fewer GFP^+^ MOp5 neurons in the right (i.e., lesioned) motor cortex, decreasing the fluorescent signal in this region and indicating a disruption in the corticospinal tract due to injury. Uninjured animals had significantly higher fluorescent signal in the right MOp (80,536 pixels/mm^3^) compared to the left MOp (33,519 pixels/mm^3^; *p* < 0.001; Figure 2I). After stroke, there was a dramatic decrease in the fluorescence intensity in the right ipsilesional MOp (to 665,2 pixels/mm^3^) compared to the right MOp of mice that received a sham surgery (*p* < 0.0001), indicating the expected loss of descending connections from the MOp to the forelimb after ischemic injury.

Previous studies found that contralesional plasticity, as detected by injections of PRV into the forelimb flexor, was associated with improved functional recovery after stroke (Liu et al., 2009). Therefore, we hypothesized that we would observe contralesional plasticity after stroke and predicted that after a focal ischemic injury, descending connections from the left contralesional MOp to the affected forelimb would increase in mice that received a stroke compared to uninjured mice. However, we did not observe any significant differences in the fluorescent signal of neuronal cell bodies compared to our control cohort (*p* = 0.1). Analysis with two-way ANOVA determined there was a significant effect of both group (stroke vs. no stroke; *p* < 0.01) and hemisphere (right vs. left; *p* < 0.01) on post-stroke neural connectivity in the MOp. Furthermore, there was a significant interaction between group and hemisphere on fluorescence intensity per mm^3^ in the MOp (*p* < 0.001).

Because neuroplasticity has also been shown in regions neighboring the ischemic lesion, we quantified changes in fluorescence intensity in the primary somatosensory cortex (SSp) (Bachmann et al., 2014). Similar to the MOp, there was a higher density of fluorescent pixels in the right SSp compared to the left SSp of uninjured mice (*p* < 0.001). Stroke diminished the fluorescent signal in the right SSp (*p* < 0.001). There was no significant difference between the left and right SSp in mice that received a stroke and no difference in fluorescence intensity in the left SSp of injured and uninjured mice.

We also quantified the fluorescent signal in neuronal processes (e.g., axons, dendrites). While the fluorescent signal in virally labeled cell bodies was brighter than that of neuronal processes, as expected, there was still robust labeling of the proximal axons and dendrites in the left and right hemisphere of mice with and without strokes. These data complement the findings observed when quantifying GFP^+^ cell bodies, showing fluorescent processes in the same regions in which we found cells (i.e., MOp5, SSp-ll). There was unilateral cortical labeling in primary motor and sensory areas, particularly in the MOp5 and SSp-ll, in uninjured mice. Fluorescent processes, predominantly apical dendrites, were identified in both the left (50,099 pixels/mm^3^) and right (95,526 pixels/mm^3^) hemisphere with significantly more labeling in the right hemisphere (Figure 2J; *p* < 0.001). In the stroke cohort, there was sparse labeling of axons in any primary motor or sensory areas, with the exception of one mouse that showed faint axonal labeling in the left contralesional MOp5 and SSp-II regions (right MOp−3,094 pixels/mm^3^). Additionally, there was moderate fluorescent signal in bilateral mid- and hindbrain nuclei in both healthy and injured mice, supporting our findings of robust labeling of neuronal cell bodies in these brain areas.

We also trained our model to identify tissue regions affected by ischemic injury. The PT model produces a small permanent focal lesion in the primary motor cortex. Fluorescent signal corresponding to damaged tissue was identified in the 3 mice that received PT strokes (Figure 2G, Supplementary Videos 7, 8). All 3 mice that received a stroke exhibited autofluorescent tissue in the MOp, our target region. Damage, albeit to a lesser extent, was also observed in the SSP-ll, a region immediately adjacent to the MOp. The control cohort of mice did not exhibit any fluorescent signal indicative of ischemic tissue in their cortical sensory and motor regions. These data confirm successful induction of stroke in the primary sensory and motor cortices.

**Figure 3:**
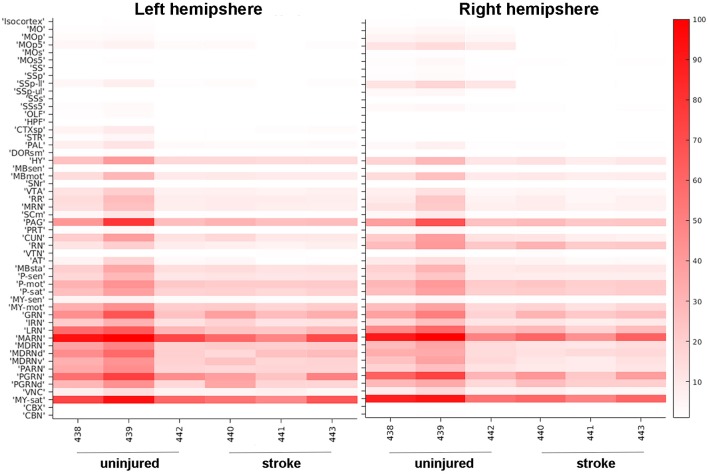
Heat map of neuronal cell body quantification in cortical and other areas. The signal intensities for pixels classified via machine learning as neuronal cell bodies in various cortical areas are shown for uninjured and stroke animals in both left hemisphere (left panel) and right hemisphere (right panel). Each brain area analyzed is listed on the left Y-axis according to the nomenclature used in the CCFv3.0 and individual animals (#, x-axis) are grouped by uninjured and stroke-injured cohorts. The intensity scale is shown on the right Y-axis and corresponds to the number of pixels classified as neuronal cell bodies normalized per region volume (pixels/mm^3^). MO, somatomotor areas; MOp, primary motor cortex; MOp5, primary motor cortex, Layer 5; MOs, supplemental motor cortex; MOs5, supplemental motor cortex, Layer 5; SS, somatosensory areas; SSp, primary somatosensory cortex; SSp-ll, primary somatosensory cortex, lower limb; SSp-ul, primary somatosensory cortex, upper limb; SSs, supplemental somatosensory cortex; SSs5, supplemental somatosensory cortex, layer 5; OLF, olfactory areas; HPF, hippocampal formation; CTXsp, cortical subplate; STR, striatum; PAL, pallidum; DORsm, thalamus, sensory motor-related; HY, hypothalamus; MBsen, midbrain, sensory-related; MBmot, midbrain, motor-related; SNr, substantia nigra, reticular part; VTA, ventral tegmental area; RR, midbrain reticular nucleus, retrorubral area; MRN, midbrain reticular nucleus; SCm, superior colliculus, motor-related; PAG, periaqueductal gray; PRT, pretectal nucleus; CUN, cuneiform nucleus; RN, red nucleus; VTN, ventral tegmental nucleus; AT, anterior tegmental area; MBsta, midbrain, behavioral state-related; P-sen, pons, sensory-related; P-mot, pons, motor-related; P-sat, pons, behavioral state-related; MY-sen, medulla, sensory-related; MY-mot, medulla, motor-related; GRN, gigantocellular reticular nucleus; IRN, intermediate reticular nucleus; LRN, lateral reticular nucleus; MARN, magnocellular reticular nucleus; MDRN, medullary reticular nucleus; MDRNd, medullary reticular nucleus, dorsal part; MDRNv, medullary reticular nucleus, ventral part; PARN, parvicellular reticular nucleus; PGRN, paragigantocellular reticular nucleus; PGRNd, paragigantocellular reticular nucleus, dorsal part; VNC, vestibular nuclei; MY-sat, medulla, behavioral state-related; CBX, cerebellar cortex; CBN, cerebellar nuclei.

In addition to detailed, region-specific visualization of connectivity in the post-stroke brain, our methodology is also able to provide global quantification of alterations in brain architecture after injury. We generated heat maps that visually represent which brain regions contain pixels classified as one of our fluorescent signals of interest, which allowed us to compare probability values between brain regions, between animals, and between the stroke and no stroke cohorts. We selected 52 brain regions to analyze, particularly focusing on regions that were associated with motor or sensory function and/or undergo dynamic changes after stroke (Bachmann et al., 2014). We also selected “control” regions that we predicted would not innervate the forelimb [e.g., hippocampal formation (HPF), olfactory areas (OLF)]. There were high fluorescent signals in many brain regions, albeit with moderate variability between mice. Fluorescence labeling from cell bodies (Figure 3) and neuronal processes (Supplementary Figure 1) was higher in the mice that did not receive a stroke. The most robust labeling for the cell bodies and neuronal processes was observed in the midbrain and hindbrain, and this labeling was largely bilateral. The brain regions with the most consistently high fluorescent signal appeared to be the medulla, behavioral state related (MY-sat) and the magnocellular reticular nucleus (MARN), a medullary motor-related nuclei, in both the uninjured (left) and injured (right) hemispheres.

### Comparison of Automated and Manual Counting

**Figure 4:**
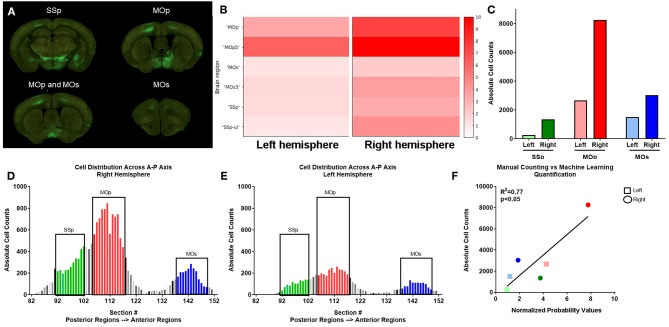
Verification of automated quantification via manual counting. **(A)** PRV labeled neurons are identified by GFP expression in serial two-photon coronal section images, including primary somatosensory (SSp), primary motor (MOp), and supplemental motor (MOs) areas. **(B)** ML identified fluorescent intensity (right y axis in heat map) in the indicated subregions (noted in the left y axis) for left and right hemispheres corresponding to the coronal sections shown in **(A)** for this uninjured mouse. Heat map show visual depiction of the summed probabilities of pixels classified as neuronal cell bodies from the ML data set, with highest intensity shown as red. **(C)** Manual quantification of PRV+ cell bodies in each region. **(D,E)** Manual quantification of PRV+ cell bodies in all coronal sections with PRV+ cell bodies for a representative uninjured brain, shown in a posterior to anterior orientation (left to right on the graph) for SSp (green), MOp (red), and MOs (blue) regions in both the **(D)** right and **(E)** left hemispheres. **(F)** Linear regression analysis of ML data (x axis) and manual counts (y axis) demonstrate a correlation between the two data sets.

In order to confirm that our ML algorithm had correctly classified pixels as neuronal cell bodies, we manually counted cell bodies in 70 coronal sections in one uninjured mouse (# 439, Figure 3). Several cortical regions were quantified: SSp, MOp, and supplemental motor cortex (MOs; Figure 4A). Our ML algorithm identified higher fluorescent signal in the right hemisphere of all regions examined, with the highest probability of classification in the MOp5 (Figure 4B). Manual counting confirmed that there was a greater number of GFP^+^ cell bodies in each of these ipsilesional right brain areas (Figure 4C). The largest number of cells was located in the right MOp, followed by the SSp, and lastly, the MOs (Figure 4D). Similar distributions of cells were observed in the left hemisphere, albeit with fewer cell bodies labeled (Figure 4E). There was a significant correlation between the normalized probability values obtained from our ML algorithm and our manual counting (*R*^2^ = 0.77; *p* < 0.05; Figure 4F). These data support the classification accuracy of the ML algorithm and registration to regions of interest.

### Whole Brain Visualization of Post-stroke Neuroinflammation

In addition to neural connectivity, we were also interested in whether STPT and our automated imaged analysis pipeline could quantify immune cell diapedesis after stroke. We focused on the migration patterns of cytotoxic CD8^+^ T cells within the brain. Early studies confirm migration of CD8^+^ T cells into the brain (Gelderblom et al., 2009), but with little to no examination of specific brain regions targeted by these cells outside of the area of injury. In order to examine this phenomenon, 13–15-week-old WT mice were given a left transient middle cerebral artery occlusion (tMCAo) and were randomly assigned to experimental groups that would either receive serial intravenous (i.v.) tail injections of either PBS or e450^+^-labeled CD8^+^ T cells. We confirmed the presence of viable e450^+^-labeled cells in the both spleens and cervical lymph nodes of recipient mice (Supplementary Figure 2). Flow cytometry confirmed the purity of e450^+^-labeled cells, as these cells were primarily CD8^+^ T cells that were present only in the CD8^+^ T cell recipient group shown both by percentage (Supplemental Figures 2B,C) and absolute cell number (Supplemental Figures 2D,E), but not the PBS controls.

**Figure 5:**
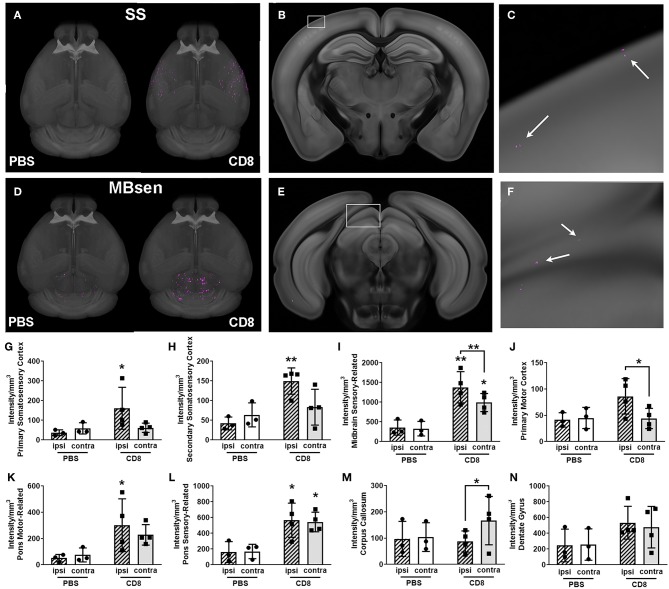
Visualization of quantification of CD8^+^ T cell diapedesis in the whole brain after stroke. **(A)** Masked 3-D probability map images of somatosensory areas only from a representative CD8^+^-treated animal (right brain) overlaid onto the CCF 3.0 average template (gray) shows pixels classified as CD8^+^ T cell (magenta) which have migrated into somatosensory areas (SS). Minimal magenta pixels are present in the PBS-treated brain (left) **(B)** Coronal section with white box highlighting part of the primary somatosensory area from the brain shown in A, with **(C)** enlargement of white boxed area showing several magenta areas classified as CD8^+^ T cells (white arrows). **(D)** 3-D probability maps from the same brains as in **(A)** but masked to show pixels classified as CD8^+^ T cells in the midbrain, sensory-related (MBsen) shows high CD8^+^ T cell diapedesis (magenta) in both hemispheres, though PBS-treated mice (left brain) also have some background level of pixels classified as fluorescently labeled CD8^+^ T cells. **(E)** Coronal section with white box highlighting MBsen from the brain shown in C, with **(F)** enlargement of white boxed area showing several magenta areas classified as CD8^+^ T cells (white arrows). **(G–N)** Bar graph shows ipsilesional (hatched bars) and contralesional (plain bars) diapedesis into selected regions in CD8^+^-treated mice (black squares, right columns) and PBS controls (black circles, left columns). Ipsilesional CD8^+^ T cell diapedesis also occurs in **(G)** primary somatosensory cortex, **(H)** supplemental somatosensory, **(I)** MBsen, **(J)** primary motor cortex, and **(I)** motor-related pons. **(L)** Sensory-related pons exhibits bilateral diapedesis, while **(M)** the corpus callosum is predominantly contralesional. **(N)** There is no significant CD8^+^ T cell diapedesis into the dentate gyrus. *p < 0.05, **p < 0.01 vs. PBS control unless otherwise indicated by brackets.

**Figure 6:**
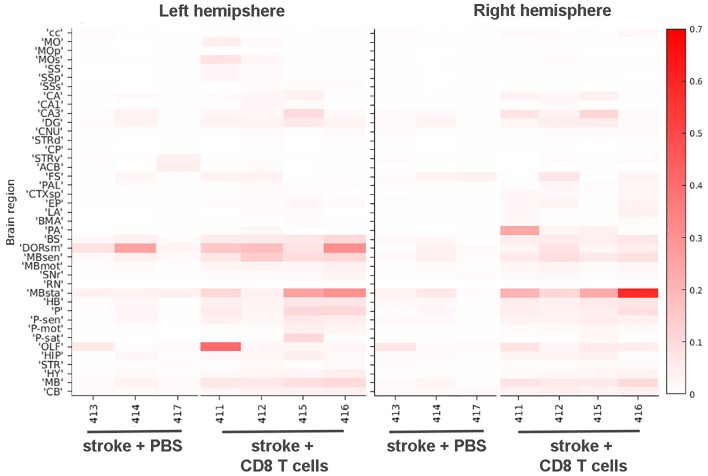
Heat map of CD8^+^ T cell diapedesis in cortical and other areas. The signal intensities for pixels classified via ML as e450-labeled CD8^+^ T cells in various cortical and other brain areas are shown for stroke animals either injected with PBS or CD8^+^ T cells in both left hemisphere (left panel) and right hemisphere (right panel). Each brain area analyzed is listed on the left Y-axis according to the nomenclature used in the CCF 3.0 and individual animals (#, x-axis) are grouped by PBS and T cell injected cohorts. The intensity scale is shown on the right Y-axis and corresponds to the number of pixels classified as CD8^+^ T cells normalized per region volume (pixels/mm^3^). cc, corpus callosum; MO, somatomotor areas; MOp, primary motor cortex; SS, somatosensory areas; SSp, primary somatosensory cortex; SSs, supplemental somatosensory cortex; CA, Ammon's horn, CA1, field CA1; CA3, field CA3; DG, dentate gyrus; CNU, cerebral nuclei; STRd, striatum, dorsal region; CP, caudate putamen; STRv, striatum, ventral region; ACB, nucleus accumbens; FS, fundus of the striatum; PAL, pallidum; CTXsp, cortical subplate; EP, endopiriform nucleus; LA, lateral amygdalar nucleus; BMA, basomedial amygdalar nucleus; PA, posterior amygdalar nucleus; BS, bed nuclei of stria terminalis; DORsm, thalamus, sensory motor-related; MBsen, midbrain, sensory-related; MBmot, midbrain, motor-related; SNr, substantia nigra, reticular part; RN, red nucleus; MBsta, midbrain, behavioral state-related; HB, hindbrain; P, pons; P-sen, pons, sensory-related; P-mot, pons, motor-related; P-sat, pons, behavioral state-related; OLF, olfactory areas; HIP, hippocampal region; STR, striatum, HY, hypothalamus, MB, midbrain, CB, cerebellum.

We trained the ML algorithm to identify e450^+^ fluorescent cells in representative sections throughout the whole brain, and observed CD8^+^ T cells within the brain tissue, traveling through blood vessels, and interestingly, along the meninges (Supplementary Videos 9, 10). CD8^+^ T cell diapedesis occurred throughout the brain, including ipsilesional somatosensory cortices (SS; Figures 5A–C) and sensory-related midbrain regions (MBsen; Figures 5D–F). 3-D renderings of our probability maps and associated videos were generated, demonstrating the bilateral movement of cells throughout the whole brain. After applying the ML algorithm to the entire cohort, a heat map was generated with 41 brain regions that were pre-selected as regions of interest for immune cell diapedesis (Figure 6). Similar to the heat maps in the neural connectivity studies (Figure 3), these heat maps allow for the comparison of the fluorescence intensity of e450^+^-labeled CD8^+^ T cells across multiple brain regions, between groups and animals. As expected, the PBS group showed low fluorescent signal in many of the brain regions, although “background” fluorescence was detected, potentially due to increased autofluorescence in dead or injured ischemic tissue secondary to the tMCAo model. Additionally, some variability was observed in the amount of CD8^+^ diapedesis between animals of the T cell-recipient group, with high diapedesis into two brain regions in particular, the behavioral state-related midbrain (MBsta) and sensory motor-related thalamus (DORsm). Our ML algorithm was not trained to detect ischemic tissue in these animals.

Overall, CD8^+^ T cells migrated into many areas of the brain of all animals 4 days after stroke, with greater diapedesis apparent in the left injured hemisphere compared to the right uninjured hemisphere. CD8^+^ T cells targeted the left ipsilesional SSp and secondary somatosensory cortex (SSs), with significantly more cells detected in the left SSp compared to PBS controls (*p* < 0.05; Figure 5G) and more cells in the left SSs (*p* < 0.01; Figure 5H). The left ipsilesional MOp was also infiltrated by CD8^+^ T cells, with more cells in the ipsilesional MOp than the corresponding contralateral MOp (*p* < 0.05; Figure 5J). Given the nature of a tMCAo injury, which affects motor and sensory cortices, it is likely these cells were targeting regions damaged by the stroke. In addition to these cortical motor and sensory regions, there was bilateral movement of CD8^+^ T cells into remote brain regions associated with motor and sensory function, namely the midbrain (Figures 5D–F,I) and the pons (Figures 5K,L). One region, corpus callosum (cc), exhibited higher contralesional CD8^+^ T cell diapedesis compared to ipsilesional cc (*p* < 0.05; Figure 5M). CD8^+^ T cells did not significantly infiltrate behavioral-state related regions in either the midbrain or the pons (data not shown) or the hippocampus (Figure 5N). Our data demonstrate that 4 days following tMCAo, CD8^+^ T cells migrate into the brain, targeting both sensory- and motor-related regions of the cortex, midbrain, and pons. In some regions, such as the MBsen and P-sen, this movement is bilateral, with cells detected in both the ipsilesional and contralesional hemisphere relative to PBS controls. In other regions, including the SSp, there is preferential migration into the ipsilesional hemisphere compared to the corresponding contralesional brain regions in CD8^+^ T cell recipients. Together, these results illustrate how STPT can be utilized to quantify region-specific diapedesis of immune cells into the ischemic brain.

## Discussion

While much research has been performed on post-stroke neuroplasticity and neuroinflammation (Gelderblom et al., 2009; Bachmann et al., 2014), these studies have been limited by conventional imaging methods. STPT, a modern microscopy technique that produces large-scale, 3-D images of intact, uncleared brain tissue, is expanding the capacity to investigate brain-wide circuit mechanisms associated with brain injury, as well as models of neurological and psychiatric disorders. Our work establishes this technique as a reliable method to quantify whole brain neural connectivity and neuroinflammation after stroke to understand region-specific mechanisms of injury and repair. STPT provides many advantages over traditional slide-based methods of mesoscale brain imaging. While automated slide scanning instruments improved our ability to image large numbers of serial sections at high resolution, sectioning and mounting artifacts such as tissue folding and tearing are unavoidable using traditional sectioning methods. These artifacts lead to inaccurate 3-D reconstruction of the whole brain. Additionally, traditional sectioning studies are often limited in scope to discrete brain regions or small numbers of whole brains due to the amount of processing and imaging time needed. STPT, through its use of automated block face imaging and sectioning, overcomes these issues. Optical clearing has also become a popular method to develop 3-D models of neuronal circuits that allows one to acquire volumetric images of the whole brain without physical sectioning (Cai et al., 2019). STPT, however, uses standard perfusion fixed brains and as such avoids a potential pitfall of optical clearing: namely, morphological changes such as increases or decreases in organ size that may distort neuronal circuits in a non-uniform way and reduce the accuracy of brain wide circuit analyses. Despite these advantages, STPT is not without its limitations. One drawback of the method is that it requires tissue to have an inherent fluorescent signal. While this is compatible with viral tracers and fluorescently labeled cells as used in our study, as well as many strains of transgenic mice with expression of fluorescent reporter proteins, it does not allow for staining with traditional primary antibodies, an accessible and commonly used technique in most laboratories. Additionally, STPT is a time-consuming method for whole brain imaging. Data could be more quickly acquired using newer technologies such as high-throughput light tomography (HTLP) (Yang et al., 2018). Future studies should utilize HTLP and other volumetric imaging techniques to confirm our findings regarding changes in axonal connectivity and CD8^+^ T cell diapedesis after stroke. Another issue to consider when using STPT or any volumetric imaging strategy is the effect of stroke on atlas registration. Missing tissue or swelling around the site of injury could lead to inappropriate designation of anatomical regions. This problem is not limited to studies of stroke. Indeed, automated quantification of STPT or any type of volumetric images of mouse models of brain injury or neurodegeneration could be similarly impaired by improper tissue warping and atlas registration due to abnormal presentation of the brain tissue.

In this study, we used STPT to examine neural connectivity and neuroinflammation. In order to study of neural connectivity we utilized PRV-152, a viral retrograde tracer. Because PRV is a trans-synaptic virus, this method allows labeling from specific muscles, to spinal interneurons and motor neurons, and ultimately to motor projection neurons in the brainstem and cerebral cortex. However, because it is a trans-synaptic tracer, one downside to this method is that we cannot distinguish between first-, second-, and third-order connections to the forelimb. New viral monosynaptic tracers, such as glycoprotein-deleted rabies virus, and traditional monosynaptic chemical tracers like FastBlue could allow us to look specifically at first-order connections, although this would require direct injections into spinal cord or brain. Furthermore, PRV is a lethal virus, inducing significant cell death and neuronal dysfunction, and ultimately killing the animal (McCarthy et al., 2009). We had a 50% mortality rate in the PT stroke cohort, while all mice that received sham surgeries survived, suggesting a detrimental synergistic effect of ischemic injury and PRV-induced cytotoxicity at 6 days after peripheral intramuscular injection. The newer viral tracers have sought to reduce or eliminate this toxicity with the promising new development of a self-inactivating glycoprotein-deleted rabies virus that allows for life-long labeling of circuits (Ciabatti et al., 2017).

It is well-established that immune cells diapedese into the infarct and penumbral regions after stroke (Xie et al., 2018), but it is less clear where and when immune cells traffic into remote brain regions that support functional recovery. Furthermore, post-stroke diapedesis has never been characterized in 3-D, making this study the first whole-brain quantification of CD8 trafficking from the periphery following stroke. One of the limitations of our study is that CD8 T cells may diapedese from the ventricles and the meninges before they reach their targeted brain tissue after stroke (Benakis et al., 2018; Yanev et al., 2019). Not all of the meningeal lymphatics are removed during sample preparation, and the ventricles are adjacent to several analyzed brain regions. Thus, it is possible that these areas could be warped and registered into the atlas, giving the false positive impression of parenchymal penetration of CD8^+^ T cells when in actuality, they are traveling through the meningeal lymphatics or ventricles to another location. It may be necessary to visually inspect coronal sections on a case by case basis, particularly if larger immune cell populations are quantified in a similar manner.

Modern techniques such as STPT and other new imaging methods further our understanding of post-stroke pathology and repair. Our data demonstrate that rapid whole brain microscopy followed by large-scale automated analyses can provide new insight into neural connectivity and neuroinflammation post-stroke in an unbiased, region-specific manner that is difficult, if not impossible, with current conventional techniques. Future studies should use these techniques to further investigate neuroplasticity and neuroinflammation following stroke, as well as other diseases and injuries with global effects on the brain.

## Data Availability Statement

All datasets generated for this study are included in the manuscript/Supplementary Files. All raw data is immediately available upon request.

## Ethics Statement

The animal study was reviewed and approved by University of Texas Southwestern Medical Center Institutional Animal Care and Use Committee.

## Author Contributions

MG, KP, and DB designed the neural connectivity experiments. KP, DB, SM, XK, and SG acquired data for neural connectivity experiments. AS and VT designed the neuroinflammation experiments. US isolated CD8^+^ T cells for neuroinflammation experiments. VT and XK acquired data for neuroinflammation experiment. EP performed all stroke surgeries. AA, JM, and DR acquired all STPT data and conducted pipeline analyses. KP, DB, VT, and AS analyzed final STPT results. All authors edited the manuscript.

### Conflict of Interest

The authors declare that the research was conducted in the absence of any commercial or financial relationships that could be construed as a potential conflict of interest.

## Supplementary Material

Supplementary Figure 1 Heat map of neuronal processes quantification in cortical and other areas.

Supplementary Figure 2 Verification of live e450-labeled CD8 T cells in recipient mice.

Supplementary Video 1 Non-stroke mouse, fly-through of raw data of GFP fluorescence.

Supplementary Video 2 Non-stroke mouse, rotation of raw data of GFP fluorescence.

Supplementary Video 3 Non-stroke mouse, fly-through of probability maps for axons and soma of GFP fluorescence.

Supplementary Video 4 Non-stroke mouse, rotation of probability maps for axons and soma of GFP fluorescence.

Supplementary Video 5 Stroke mouse, fly-through of raw data of GFP fluorescence.

Supplementary Video 6 Stroke mouse, rotation of raw data of GFP fluorescence.

Supplementary Video 7 Stroke mouse, fly-through of probability maps for axons and soma of GFP fluorescence.

Supplementary Video 8 Stroke mouse, rotation of probability maps for axons and soma of GFP fluorescence.

Supplementary Video 9 Stroke mouse, fly-through of raw data of CD8 T cell fluorescence.

Supplementary Video 10 Stroke mouse, fly-through of probability map for CD8 T cell fluorescence.
