# Supplementary material for: Visualization and Quantification of Post-stroke Neural Connectivity and Neuroinflammation Using Serial Two-Photon Tomography in the Whole Mouse Brain
Source: Front Neurosci. 2019 Oct 4;13:1055. doi: 10.3389/fnins.2019.01055 (PMC6787288; doi:10.3389/fnins.2019.01055)
Supplement: Supplementary Figure 1 — Heat map of neuronal processes quantification in cortical and other areas. [file Data_Sheet_1.docx]

**Visualization and quantification of neuroplasticity and neuroinflammation using serial two-photon tomography in the whole mouse brain**

Katherine Poinsatte*,^1^ Dene Betz*,^1^ Vanessa O. Torres*,^1^ Apoorva D. Ajay,^1^ Shazia Merza,^1^ Uma M. Selvaraj,^1^ Erik J. Plautz,^1^ Xiangmei Kong,^1^ Sankalp Gokhale,^1^ Julian P. Meeks,^1,2^ Denise M. O. Ramirez,^1^ Mark P. Goldberg*,^1^ and Ann M. Stowe*^1,3^

* denotes equal contribution

^1^ Department of Neurology and Neurotherapeutics, ^2^ Department of Neuroscience, UT Southwestern Medical Center, Dallas TX, USA. ^3^Department of Neurology, University of Kentucky, Lexington, KY, USA.

**Supplementary Figures**

**
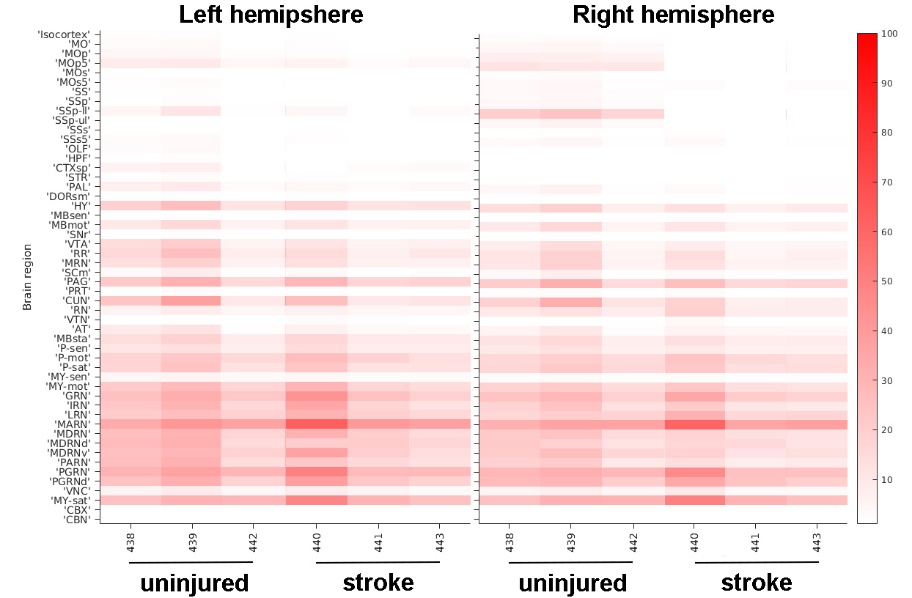
**

**Supplementary Fig. 1 Heat map of neuronal processes quantification in cortical and other areas.** The signal intensities for pixels classified via machine learning as neuronal processes in various cortical areas are shown for uninjured and stroke animals in both left hemisphere (left panel) and right hemisphere (right panel). Each brain area analyzed is listed on the left Y-axis according to the nomenclature used in the CCFv3.0 and individual animals (#, x-axis) are grouped by uninjured and stroke-injured cohorts. The intensity scale is shown on the right Y-axis and corresponds to the number of pixels classified as neuronal cell bodies normalized per region volume (pixels/mm^3^). MO – somatomotor areas; MOp – primary motor cortex; MOp5 – primary motor cortex, Layer 5; MOs – secondary motor cortex; MOs5 – secondary motor cortex, Layer 5; SS – somatosensory areas; SSp – primary somatosensory cortex; SSp-ll – primary somatosensory cortex, lower limb; SSp-ul – primary somatosensory cortex, upper limb; SSs – secondary somatosensory cortex; SSs5 – secondary somatosensory cortex, layer 5; OLF – olfactory areas; HPF – hippocampal formation; CTXsp – cortical subplate; STR – striatum; PAL – pallidum; DORsm – thalamus, sensory motor-related; HY – hypothalamus; MBsen – midbrain, sensory-related; MBmot – midbrain, motor-related; SNr – substantia nigra, reticular part; VTA – ventral tegmental area; RR – midbrain reticular nucleus, retrorubral area; MRN – midbrain reticular nucleus; SCm – superior colliculus, motor-related; PAG – periaqueductal gray; PRT – pretectal nucleus; CUN – cunieform nucleus; RN – red nucleus; VTN – ventral tegmental nucleus; AT – anterior tegmental area; MBsta – midbrain, behavioral state-related; P-sen – pons, sensory-related; P-mot – pons, motor-related; P-sat – pons, behavioral state-related; MY-sen – medulla, sensory-related; MY-mot – medulla, motor-related; GRN – gigantocellular reticular nucleus; IRN – intermediate reticular nucleus; LRN – lateral reticular nucleus; MARN – magnocellular reticular nucleus; MDRN – medullary reticular nucleus; MDRNd – medullary reticular nucleus, dorsal part; MDRNv – medullary reticular nucleus, ventral part; PARN – parvicellular reticular nucleus; PGRN – paragigantocellular reticular nucleus; PGRNd – paragigantocellular reticular nucleus, dorsal part; VNC – vestibular nuclei; MY-sat – medulla, behavioral state-related; CBX – cerebellar cortex; CBN – cerebellar nuclei

**
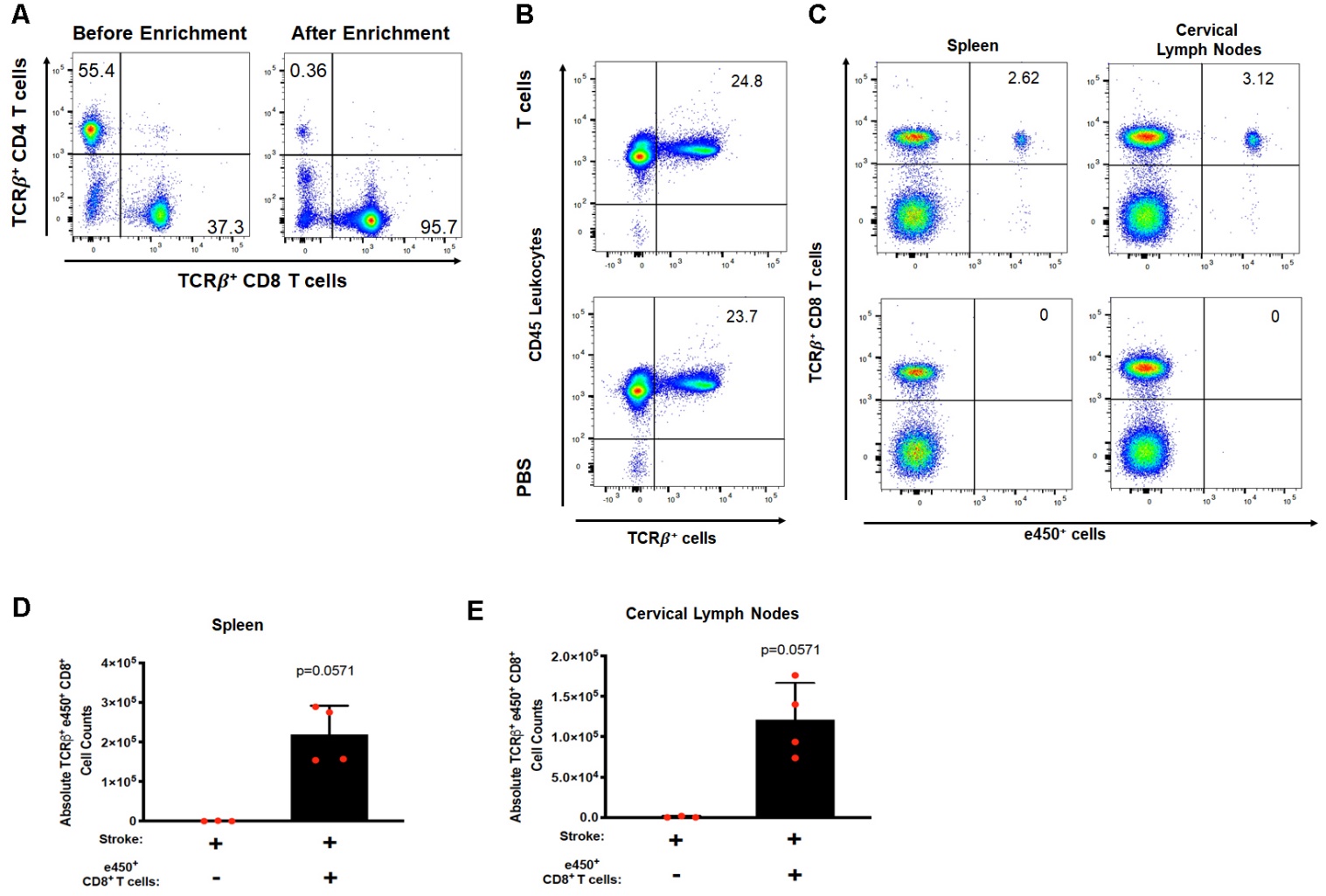
**

**Supplementary Fig. 2 Verification of live e450-labeled CD8 T cells in recipient mice.** (A) Flow cytometry plots for CD8+ T cells (x axis) vs. CD4 T cells (y axis) shows purification of the CD8 T cell population for adoptive transfer. (B) Flow plots show CD8 T cell populations (x axis) from CD45+ leukocyte populations (y axis) for e450+ CD8 cell-treated (top row) and PBS-treated (bottom row) mice. (C) CD8+ T cells (y axes) labeled with e450 (x axes) are only in the spleens and cervical lymph nodes (CLN) of T cell recipient mice (top row). Quantification of cell numbers show e450+ CD8 T cells only in the (D) spleen and (E) cervical lymph nodes in recipient mice (right columns).
